# Ets-1 Regulates Energy Metabolism in Cancer Cells

**DOI:** 10.1371/journal.pone.0013565

**Published:** 2010-10-22

**Authors:** Meghan L. Verschoor, Leigh A. Wilson, Chris P. Verschoor, Gurmit Singh

**Affiliations:** 1 Department of Research, Juravinski Cancer Centre, Hamilton, Ontario, Canada; 2 Department of Medical Science, McMaster University, Hamilton, Ontario, Canada; 3 Department of Pathology and Molecular Medicine, McMaster University, Hamilton, Ontario, Canada; Duke University, United States of America

## Abstract

Cancer cells predominantly utilize glycolysis for ATP production even in the presence of abundant oxygen, an environment that would normally result in energy production through oxidative phosphorylation. Although the molecular mechanism for this metabolic switch to aerobic glycolysis has not been fully elucidated, it is likely that mitochondrial damage to the electron transport chain and the resulting increased production of reactive oxygen species are significant driving forces. In this study, we have investigated the role of the transcription factor Ets-1 in the regulation of mitochondrial function and metabolism. Ets-1 was over-expressed using a stably-incorporated tetracycline-inducible expression vector in the ovarian cancer cell line 2008, which does not express detectable basal levels of Ets-1 protein. Microarray analysis of the effects of Ets-1 over-expression in these ovarian cancer cells shows that Ets-1 up-regulates key enzymes involved in glycolysis and associated feeder pathways, fatty acid metabolism, and antioxidant defense. In contrast, Ets-1 down-regulates genes involved in the citric acid cycle, electron transport chain, and mitochondrial proteins. At the functional level, we have found that Ets-1 expression is directly correlated with cellular oxygen consumption whereby increased expression causes decreased oxygen consumption. Ets-1 over-expression also caused increased sensitivity to glycolytic inhibitors, as well as growth inhibition in a glucose-depleted culture environment. Collectively our findings demonstrate that Ets-1 is involved in the regulation of cellular metabolism and response to oxidative stress in ovarian cancer cells.

## Introduction

Over 50 years ago, Otto Warburg first proposed that mitochondrial injury that leads to depressed electron transport chain function and respiratory defects is an important step in the development and progression of carcinogenesis [1], [2]. Over the past two decades, many studies have shown that tumours preferentially use glycolysis for energy production over oxidative phosphorylation, a characteristic that has become a hallmark of tumour pathophysiology [3]–[5]. Known as the Warburg effect, cancer cells predominantly utilize glycolysis for ATP production even in the presence of abundant oxygen, an environment that would normally result in energy production through oxidative phosphorylation [2], [6]. Alterations in mitochondrial DNA correspond to increased production of ROS and impaired oxidative phosphorylation, resulting in decreased ATP production and increased glycolytic dependence [5], [7]. The increased production of reactive oxygen species, particularly H_2_O_2_, by cancer cells is likely the result of mitochondrial dysfunction, as mitochondria are considered to be the predominant cellular source of reactive oxygen species [8]. Four to five percent of the O_2_ consumed by oxidative phosphorylation in the mitochondria is normally converted to reactive oxygen species; therefore defects to the electron transport chain system in cancer cells would result in excessive reactive oxygen species formation [8]–[10]. Excessively produced, H_2_O_2_ can act as a signaling molecule by oxidizing cysteine molecules on proteins, activating several signaling pathways including MAPK, as well as several transcription factors including AP-1, p53, NF-κB, and Ets-1 [11]–[15].

Ets-1, a member of the Ets protein family of transcription factors, regulates the expression of a diverse set of proteins through its interaction with specific consensus sequences upstream of target genes [16]. The over-expression of Ets-1 has been associated with a multitude of different cancers, specifically with regards to tumour progression and invasion [16]–[19]. Additionally, over-expression of Ets-1 has also been associated with poor prognosis in breast [20], ovarian [21], and cervical carcinomas [22]. Traditionally, Ets-1 is thought to function as a transcriptional activator and its high expression in endothelial and stromal cells correlates with tumour cell invasiveness and unfavourable outcome in ovarian and breast cancer [23]–[25]. Our laboratory and that of others have highlighted the importance of Ets-1 in the regulation of different aspects of cancer cell behaviour, including extracellular matrix remodeling, invasion, angiogenesis [26], and drug resistance [27]. The link between Ets-1 and cancer invasiveness can potentially be explained by the list of known target genes regulated by this transcription factor. Several MMPs and integrin genes, as well as urokinase plasminogen activator (uPA), which are all known mediators of extracellular matrix degradation and cell migration, are known targets for Ets-1 [28]–[31]. Many genes crucial to angiogenesis and extracellular matrix remodeling, such as matrix metalloproteinases (MMP-1, MMP-3, MMP-9), uPA and Integrinβ3, are under the direct regulation of Ets-1 [26]. This transcription factor is thus considered to be an important mediator of cancer cell development and tumour progression.

In this study we have demonstrated that Ets-1 plays a role in the regulation of energy metabolism in ovarian cancer cells. Using high throughput genomic analysis, we have found that Ets-1 regulates, either directly or indirectly, several important genes involved in mitochondrial metabolic and antioxidant defense pathways in our Ets-1 over-expression ovarian cancer cell model. Functionally, we have shown that glycolysis, oxidative phosphorylation, and cellular respiratory systems are altered in response to changes in Ets-1 expression. Taken together, our findings indicate that Ets-1 is a key transcription factor involved in regulating metabolic and oxidative stress in cancer cells.

## Results

### Cancer cell model of Ets-1 gene expression

**Figure 1:**
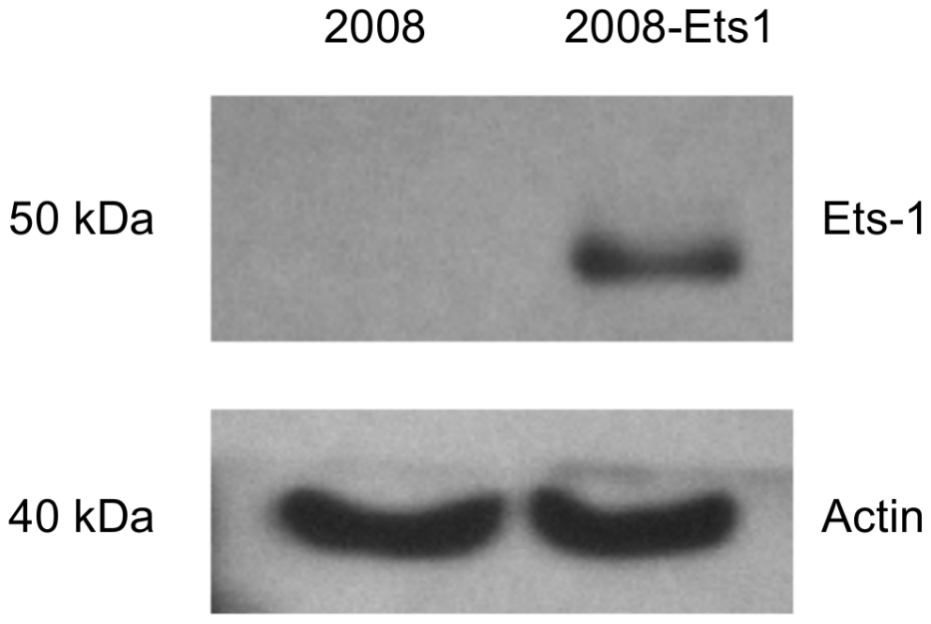
Generation of an ovarian cancer cell model for Ets-1 expression. 2008 ovarian cancer cells were stably transfected to over-express Ets-1 in a tetracycline inducible system. Protein expression of 2008 and 2008-Ets1 cells was measured via Western blot following induction with tetracycline (n = 3).

**Figure 2:**
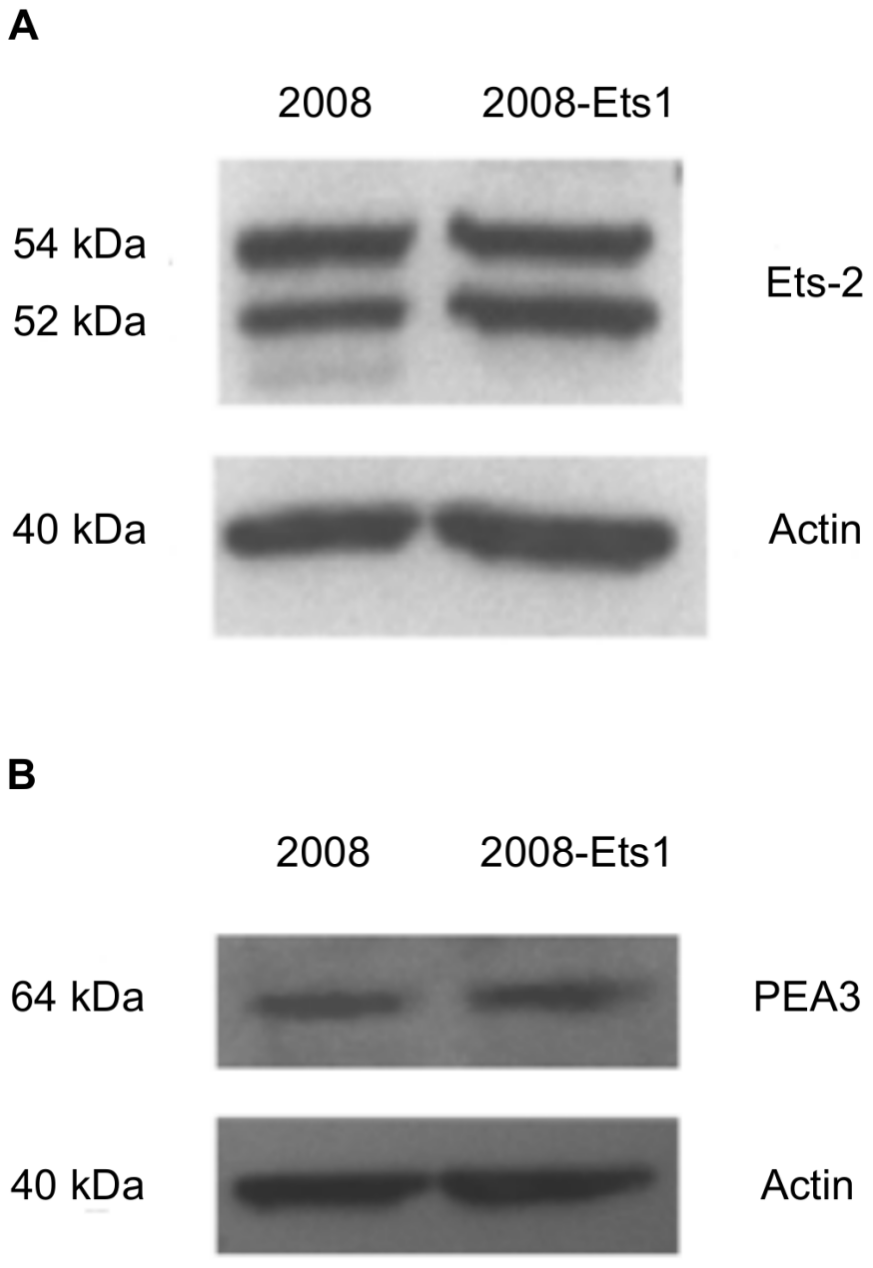
Ets-1 expression did not affect the expression of similar ETS family members. The protein expression of ETS family transcription factors (**A**) Ets-2 and (**B**) PEA3 were compared in 2008 and 2008-Ets1 ovarian cancer cells to determine the specificity of our Ets-1 expression model. Western blot analysis showed that neither of these transcription factors was affected by Ets-1 expression in 2008 cells.

Ets-1 was over-expressed in 2008 ovarian cancer cells using a tetracycline-inducible system, generating 2008-Ets1 cells. Ets-1 protein expression in tetracycline-treated 2008 and 2008-Ets1 was examined via Western blotting (Figure 1). Ets-1 protein expression was not detectable in 2008 whole cell lysates, but was readily detected in the induced 2008-Ets1 lysate. Increased Ets-1 expression was found to be a specific effect as the protein levels of two similar Ets family members, Ets-2 and PEA3 were not altered in this model of Ets-1 over-expression (Figure 2).

### Ets-1 regulates metabolic gene expression

Microarray analysis of 2008 and 2008-Ets1 cells revealed that 3,131 genes of the 28,869 genes probed were up-regulated or down-regulated in response to Ets-1 over-expression by at least 1.45 fold (p≤0.001) (GEO database accession #GSE21129). For this study, we have chosen to report and examine changes in selected mRNAs whose functions are relevant to mitochondrial activity, cellular metabolism, and oxidative stress. Real time qRT-PCR validation was conducted using 10 target genes representing various fold change values, and results were normalized to 4 separate housekeeping genes. Although both PPARG and SDHB gene expression in 2008-Ets1 cells were not significantly different from 2008 cells, the fold changes determined from real time qRT-PCR were associated with the microarray fold changes by a correlation coefficient of 0.99 (Table 1). Therefore, fold changes greater than 1.45 were deemed to be valid results and were included in this study.

**Table 1:** Real time qRT-PCR validation of microarray findings.

Gene name	Microarray fold change	Real time PCR fold change	Valid?
ETS1	12.70 ***	10.14 **	Y
GPX2	8.80 ***	9.95 ***	Y
PPARG	2.58 ***	2.07	N
G6PD	1.74 ***	1.91 *	Y
HK1	1.71 ***	2.18 **	Y
SDHB	−1.30 ***	−2.07	N
CYC1	−1.46 ***	−2.87 *	Y
PDHA	−2.05 ***	−1.99 *	Y
NDUFAB1	−2.23 ***	−2.76 ***	Y
MMP13	−11.00 ***	−11.26 ***	Y

Correlation Coefficient = 0.99; p≤0.05 = *, p≤0.01 = **, p≤0.001 = ***.

The expression of G6PD, PDHA, HK, and CYC were examined by Western blotting to determine whether the gene expression differences observed were also present at the protein level. We did not find any significant differences in protein expression between 2008 and 2008-Ets1 cells for any of the enzymes examined (data not shown).

### The glycolytic capability of cancer cells is regulated by Ets-1

**Figure 3:**
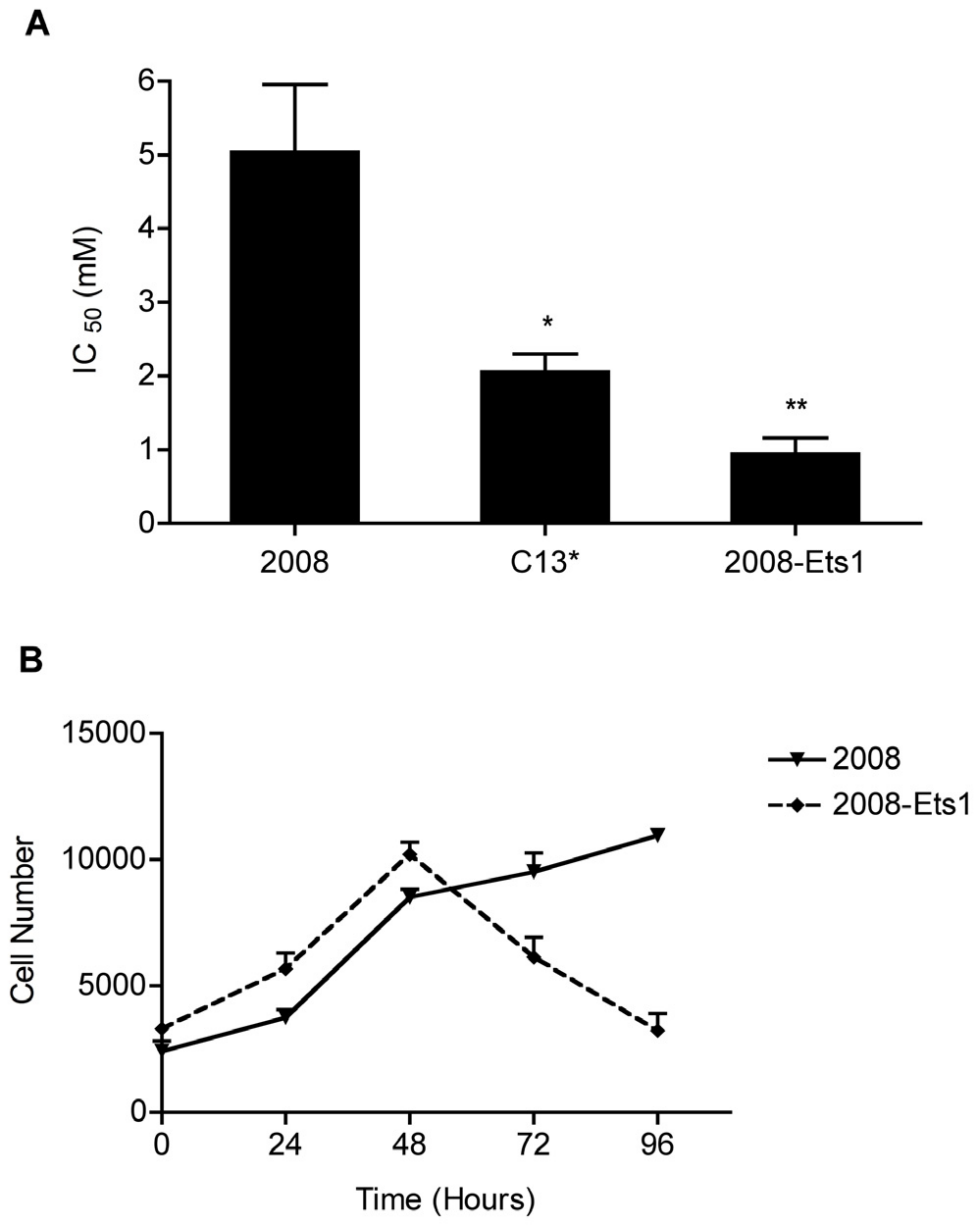
Glycolytic dependence of Ets-1-expressing ovarian cancer cells. (**A**) 2008, C13*, and 2008-Ets1 cells were treated with the glycolytic inhibitor 2-DG, and cellular growth was measured following 96 hours of incubation. 2008-Ets1 and C13* cells which express Ets-1 showed significantly decreased growth following glycolytic inhibition in 2008-Ets1 compared to parental 2008 cells (n = 4). (**B**) 2008 and 2008-Ets1 cells were grown in the absence of glucose, and proliferation assays were conducted at 24 hour intervals. 2008-Ets1 ovarian cancer cells expressing high levels of Ets-1 showed a decreased ability for growth in glucose-free culture medium, suggesting an increased reliance on glycolysis for energy (n = 3).

Microarray analysis of 2008-Ets1 ovarian cancer cells revealed that Ets-1 is involved in the regulation of mitochondrial stress and dysfunction as metabolic genes, including those involved in glycolysis, glycolytic feeder pathways, the TCA cycle, and lipid metabolism (Table 2), and genes involved in antioxidant defense (Table 3) were altered in Ets-1 over-expressing cells. To evaluate whether cells with stable over-expression of Ets-1 favour glycolysis over oxidative phosphorylation for energy, as predicted by our microarray analysis, cells were grown in glucose-free media supplemented with the glycolytic inhibitor 2-DG, an analog of glucose. Cells were grown in the presence of varying amounts of 2-DG, and representative growth curves were generated for each cell line. The growth of cells in media containing 2-DG was inhibited to a greater extent in cells with a higher Ets-1 expression than in parental cells (Figure 3A). The 2-DG IC_50_ doses, or doses where 50% of the cells had stopped proliferating, were calculated from 4 independent experiments. Our results indicated that 2008-Ets1 cells induced with tetracycline were the most sensitive to 2-DG, with an IC_50_ of 0.75 mM, which was significantly lower than the parental 2008 cells, IC_50_ of 4.29 mM (p≤0.01). C13* cells, a cisplatin-resistant variant of 2008 cells, had an IC_50_ of 2.04 mM, which was also significantly lower than parental 2008 cells (p≤0.05). To discount any treatment effect, parental 2008 cells were treated with tetracycline, and showed no significant growth inhibition by 2-DG with an IC_50_ of 3.67 mM (data not shown). Growth of cells in normal or glucose-free media (supplemented with sodium pyruvate) was compared over 96 hrs, after which it was observed that the proliferation of cells with increased expression of Ets-1 was notably slower compared to parental 2008 cells (Figure 3B). Treatment with glucose-free media resulted in a decreased proliferation rate for all cells tested in comparison to normally supplemented media (data not shown).

**Table 2:** Effect of Ets-1 over-expression on glycolysis, glycolytic feeder pathways, and lipid metabolism.

Pathway	Gene	GenBank ID	Gene name	Fold change
Glycolysis	Enolase 2	NM_001975	ENO2	+2.38 ***
	Hexokinase1	NM_033500	HK1	+1.71 ***
	Aldolase C, fructose-bisphosphate	NM_005165	ALDOC	+2.66 ***
	Phosphoglucomutase 1	NM_002633	PGM1	+1.59 ***
Galactose	UDP-galactose-4-epimerase	NM_000403	GALE	+1.57 ***
	Galactose mutarotase (aldose 1-epimerase)	NM_138801	GALM	−3.54 ***
Glycogen	Phosphorylase, glycogen, liver	NM_002863	PYGL	+2.00 ***
Citric acid cycle	Citrate synthase	NM_004077	CS	−1.63 ***
	Fumarate hydratase	NM_000143	FH	−1.48 ***
Pentose phosphate	Glucose-6-phosphate dehydrogenase	NM_000402	G6PD	+1.74 ***
	Phosphoglucomutase 1	NM_002633	PGM1	+1.59 ***
	Transketolase	NM_001135055	TKT	+2.07 ***
Lipid metabolism	Apolipoprotein B mRNA editing catalytic polypeptide-like 3G	NM_021822	APOBEC3G	+2.25 ***
	Fatty acid desaturase 1	NM_013402	FADS1	+6.47 ***
	Fatty acid desaturase 2	NM_004265	FADS2	+5.29 ***
	Fatty acid synthase	NM_004104	FASN	+1.61 ***
	Lysophosphatidylcholine acyltransferase 2	NM_017839	LPCAT2	+2.56 ***
	Phospholipase C, beta 3	NM_000932	PLCB3	+2.75 ***
	Prostaglandin-endoperoxide synthase 1	NM_000962	PTGS1	+3.88 ***
	Protein kinase, AMP-activated, beta 2	NM_005399	PRKAB2	+2.05 ***

p≤0.05 = *, p≤0.01 = **, p≤0.001 = ***.

**Table 3:** Effect of Ets-1 over-expression in oxidative stress.

Pathway	Gene	GenBank ID	Gene name	Fold change
Antioxidant defense	Glutathione peroxidase 1	NM_000581.2	GPX1	+1.98 ***
	Glutathione peroxidase 2	NM_005333	GPX2	+8.81 ***
	Glutathione peroxidase 3	NM_002084	GPX3	+4.19 ***
	Peroxiredoxin 5	NM_012094	PRDX5	+1.54 ***
ROS metabolism	Arachidonate 12-lipoxygenase	NM_000697	ALOX12	−1.81 **
	GTF2I repeat domain containing 2	NM_173537	GTF2IRD2	+1.92 ***
	GTF2I repeat domain containing 2B	NM_001003795	GTF2IRD2B	+2.19 ***
	Neutrophil cytosolic factor 2	NM_000433	NCF2	−2.24 ***
	Superoxide dismutase 2 (mitochondrial)	NM_001024465	SOD2	−1.49 ***
	ATX1 antioxidant protein 1	NM_004045	ATOX1	+1.53 ***
	Prion protein	NM_000311	PRNP	−1.60 ***
	Scavenger receptor class A, member 3	NM_016240	SCARA3	+1.48 **
	Selenoprotein P, plasma, 1	NM_005410	SEPP1	−2.04 ***
	Aldehyde oxidase 1	NM_001159	AOX1	+2.81 ***
	BCL2/adenovirus E1B interacting protein 3	NM_004052	BNIP3	+3.15 ***

p≤0.05 = *, p≤0.01 = **, p≤0.001 = ***.

### Ets-1 regulates cellular oxygen consumption in cancer cells

**Figure 4:**
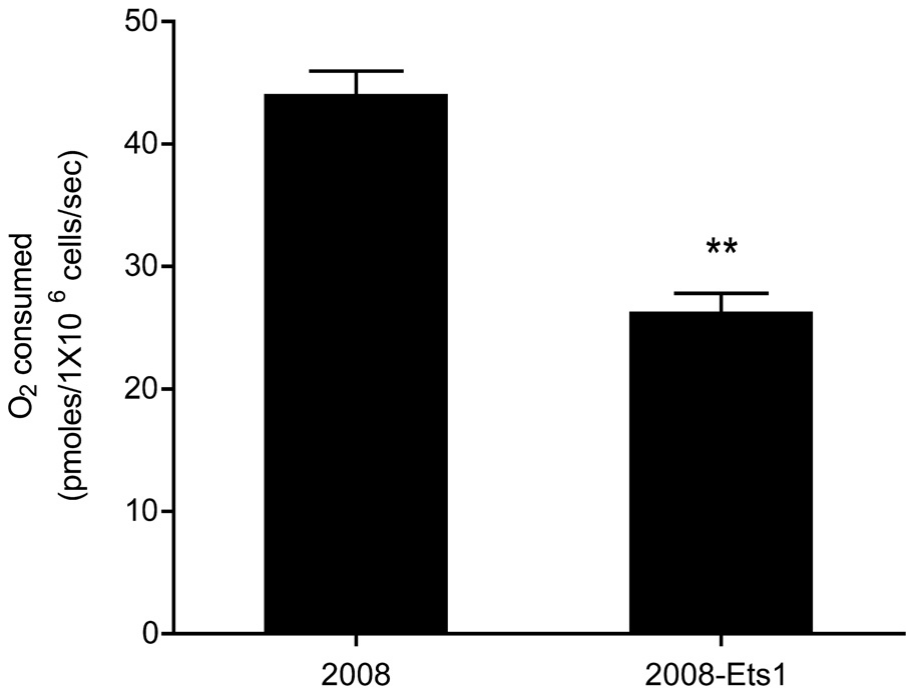
Effect of Ets-1 expression on oxygen consumption. Basal oxygen consumption was determined for 2008 and 2008-Ets1 ovarian cancer cells. Ets-1 expression was associated with a significant decrease in basal oxygen consumption suggesting a decreased reliance on oxidative phosphorylation in 2008-Ets1 cells.

Our microarray analyses suggest that Ets-1 over-expression resulted in an overall down-regulation of genes that encode electron transport chain components, suggesting that these cell lines would likely display decreased O_2_ consumption (Table 4). This assumption was evaluated using high-resolution respirometry, where basal oxygen consumption was measured following the addition of cells to an oxygraph. Basal oxygen consumption was significantly lower in induced 2008-Ets1 cells (26.23 pmoles O_2_/1×10^6^ cells/sec; p≤0.05) compared to 2008 cells (40.60 pmoles O_2_/1×10^6^ cells/sec, p≤0.05) (Figure 4). No significant tetracycline treatment effect on basal oxygen consumption was found following induction of 2008 cells, confirming that tetracycline did not affect oxygen consumption in our model (data not shown).

**Table 4:** Effect of Ets-1 over-expression on the ETC.

Component	Gene	GenBank ID	Gene name	Fold change
Complex I	NADH dehydrogenase (ubiquinone) 1 α subcomplex 1	NM_004541	NDUFA1	−1.60 ***
	NADH dehydrogenase (ubiquinone) 1 α subcomplex 9	NM_005002	NDUFA9	−1.59 ***
	NADH dehydrogenase (ubiquinone) 1, α/β subcomplex 1	NM_005003	NDUFAB1	−2.23 ***
	NADH dehydrogenase (ubiquinone) 1 beta subcomplex, 5	NM_002492	NDUFB5	−1.50 ***
	NADH dehydrogenase (ubiquinone) 1 β subcomplex 11	NR_024234	NDUFB11	−1.48 ***
	NADH dehydrogenase (ubiquinone) 1 α subcomplex assembly factor 1	NM_016013	NDUFAF1	−1.71 ***
	NADH dehydrogenase (ubiquinone) Fe-S protein 4	NM_002495	NDUFS4	−1.58 ***
Complex III	Cytochrome *c*-1	NM_001916	CYC1	−1.46 ***
	Ubiquinol-cytochrome *c* reductase, Rieske iron-sulfur polypeptide 1	NM_006003	UQCRFS1	−1.50 ***
	Ubiquinol-cytochrome *c* reductase, complex III subunit VII	NM_014402	UQCRQ	−1.50 ***
Complex IV	Cytochrome *c* oxidase subunit 7b	NM_001866	COX7B	−2.08 ***
ATP Synthases	ATP synthase, H+ transporting, mitochondrial F1 complex, α subunit 1, cardiac muscle	NM_001001937	ATP5A1	−1.53 ***
	ATPase, H+ transporting, lysosomal accessory protein 2	NM_005765	ATP6AP2	−1.50 ***
Electron transport associated factors	Cytochrome *c*, somatic	NM_018947	CYCS	−1.51 ***
	Holocytochrome c synthase (cytochrome c heme-lyase)	NM_005333	HCCS	−1.76 ***

p≤0.05 = *, p≤0.01 = **, p≤0.001 = ***.

## Discussion

Mitochondrial reactive oxygen species activate several key signaling pathways involved in tumourigenesis and up-regulate the expression of important oncogenic transcription factors including Ets-1 [32]. We have previously shown that increased production of intracellular reactive oxygen species in C13* cells, a cisplatin-resistant variant of 2008 ovarian carcinoma cells, correlated with an increase in Ets-1 mRNA and protein expression [15], [33]. Subsequent treatment of these cells with H_2_O_2_ increased Ets-1 expression in a dose-dependent manner, suggesting that this transcription factor is highly responsive to tumour-derived signals.

Our previous analysis of the Ets-1 promoter led to the identification of an antioxidant response element (ARE) that proved to be pivotal in regulating the expression of Ets-1 under both basal and H_2_O_2_-induced conditions [15]. Traditionally, the functional importance of Ets-1 over-expression in cancer has been associated with the regulation of matrix-degrading proteases and angiogenic factors [26], [34]. However, our recent findings that mitochondrial reactive oxygen species potently affects Ets-1 expression at the transcriptional level [15] suggest that the importance of this transcription factor in cancer initiation and progression extends beyond angiogenesis and metastasis alone.

We hypothesized that Ets-1 may be involved in the regulation of mitochondrial metabolism in cancer cells because mitochondrial stress from both increased reactive oxygen species production and electron transport chain malfunction result in increased Ets-1 mRNA and protein [15]. In order to determine the functional importance of Ets-1 expression in cancer cell metabolism, we generated the tetracycline-inducible Ets-1 over-expressing ovarian cancer cell line 2008-Ets1. Parental 2008 cells do not express detectable levels of Ets-1 protein endogenously. To analyze the genomic consequences of Ets-1 over-expression in these ovarian cancer cells, we conducted a human gene microarray. Our findings indicate that Ets-1 is either directly or indirectly involved in regulating the expression of more than 3,000 of the over 28,000 human genes examined. Interestingly, our findings suggest that Ets-1 could act as a transcriptional repressor of more than half (1,665) of these genes in ovarian carcinoma cells.

Although Ets-1 has been studied extensively in both physiological and pathological processes [17], it is rarely referred to as a transcriptional repressor. In the context of mitochondrial dysfunction and metabolism, Ets-1 was found to at least partially down-regulate several components of the electron transport chain. Complex I, the most prominent site for electron leakage leading to excessive reactive oxygen species production in the electron transport chain, is composed of 39 nuclear encoded subunit genes, of which Ets-1 down-regulates 11. Another important site for electron leakage and reactive oxygen species production is Complex III, which is composed of 10 subunits, of which Ets-1 down-regulates 3. Ets-1 also represses components of Complex IV, Complex V ATP synthases, as well as some electron transport associated factors. Taken together, these repressive functions suggest that Ets-1 is prominently involved in the decreased reliance on oxidative phosphorylation frequently associated with cancer cells. Consequently, genes encoding mitochondrial proteins involved in oxidative phosphorylation would be down-regulated, and cells would require alternate methods of ATP production including glycolysis and fatty acid oxidation. Given that components of almost every complex of the electron transport chain, several key enzymes of the TCA cycle, and ultimately the reducing equivalents needed for electron transport were similarly down-regulated, Ets-1 over-expressing cells appear to have a decreased capacity to generate ATP via oxidative phosphorylation at the gene expression level.

Cancer cells commonly have a decreased reliance on oxidative phosphorylation for energy generation, and they likewise have an increased dependence on glycolysis and fat metabolism for cellular energy [35]. In further support that Ets-1 is involved in the regulation of altered cancer metabolism, Ets-1 is associated with the increased expression of many genes involved in glycolysis, glycolytic feeder pathways, and the pentose phosphate pathway. Additionally, the expression of Ets-1 is also correlated with increases in the expression of many genes involved with lipid metabolism and biosynthesis. Taken together, these trends suggest that Ets-1 is an important transcriptional regulator not only in the catabolic, but also anabolic metabolic transitions of cancer cells that ultimately promote tumourigenesis [35].

It was almost half a century ago when the up-regulation of fatty acid synthase (FASN) was first described in cancer [36], which is now known to be over-expressed in the majority of cancers. Interestingly, Ets-1 was also found to be involved in the regulation of increased FASN gene expression in our ovarian cancer model. Thus, our gene expression findings suggest that Ets-1 is a key transcription factor involved in the metabolism of cancer cells, and particularly important in the metabolic shift towards glycolysis and anabolic means of energy production. Although it is important to note that Ets-1-mediated metabolic regulation is likely achieved by a large consortium of different transcription factors, a more complex regulatory network of transcription factors influenced by mitochondrial dysfunction have yet to be elucidated. We have demonstrated that the over-expression of Ets-1 in our ovarian cancer model did not affect protein levels of two closely related ETS family members; however, it is possible that other ETS transcription factors from this very large family also influence cancer metabolism. Considering that these transcription factors recognize almost identical consensus sequences, the repetition of the experiments within this study following the over-expression of other ETS family members could yield similar results. In addition, such experiments would further characterize the potentially large transcriptional network involved in the specialized metabolism of cancer cells.

To determine the functional relevance of our genomic results, we have examined the glycolytic capability of our Ets-1 over-expression model. We have indirectly evaluated the oxidative phosphorylation capacity of these cells through treatment with the glycolytic inhibitor 2-DG. Ovarian C13* and induced 2008-Ets1 cells, which both express Ets-1, showed prominent growth inhibition in response to 2-DG, suggesting that these cells are more reliant on glycolysis for ATP production. Additionally, the Ets-1 over-expressing ovarian cancer cells displayed significantly decreased growth following glucose deprivation, further emphasizing their glycolytic reliance. The growth rate of all cell types in glucose-free media was decreased as compared to normally supplemented media, particularly after 48 hrs when a distinct divergence in cell growth was consistently observed, likely due to decreased glucose availability. Over-expression of Ets-1 potently exacerbated this divergence as the growth of induced 2008-Ets1 cells drastically decreased after 48 hrs. However at the protein level, we did not find any significant differences in the expression of glucose-6-phosphate dehydrogenase, pyruvate dehydrogenase, cytochrome *c*, or hexokinase, which are all enzymes involved in glycolysis or oxidative phosphorylation. Importantly, we did see repeatable and significant differences in glycolytic dependence associated with Ets-1 expression, and the lack of changes in protein expression could therefore be due to post-translational modifications mediated by Ets-1. For example, differences within the catalytic region of an enzyme would result in functional differences, but would not necessarily be detectable via Western blot, as this technique is dependent on the specific epitope targeted by the antibody used. Thus, we plan to examine the enzyme activity of these metabolic enzymes in future studies to determine if any differences exist between 2008 and 2008-Ets1 cells that could account for the functional differences we have demonstrated in this study.

The evaluation of O_2_ consumption of a population of cells is a functional evaluation of oxidative capacity, as it represents a good estimate of the rate at which electrons are passing along the electron transport chain and being reduced to H_2_O_2_. The polarographic system used in this study to measure O_2_ consumption includes sensors that yield a current proportional to the partial pressure of O_2_ in cell containing media by consuming O_2_ in a cathode half reaction, thus the signal responds exponentially to changes in O_2_ pressure within the sample. In the absence of specific complex substrates and ADP, thereby simulating respiration, the basal rate of O_2_ consumption can be measured. We have observed significant decreases in O_2_ consumption in cells with increased Ets-1 expression. Our results indicate that Ets-1 is directly involved in the regulation of cellular oxidative capacity, where Ets-1 over-expression led to significantly decreased O_2_ consumption, and Ets-1 down-regulated cells displayed a very prominent increase in O_2_ consumption. These results suggest that up-regulated Ets-1 expression promotes a decreased dependence on oxidative phosphorylation for energy, and provides further evidence towards the functional importance of Ets-1 in cancer cell metabolism.

High levels of oxidative stress are typically observed in the tumour microenvironment as a result of imbalances in antioxidant defense factors, and impaired DNA repair mechanisms [37]–[39]. In breast cancer cells lines, increased malignancy is associated with high levels of reactive oxygen species-producing superoxide dismutase activity, in combination with decreased levels of reactive oxygen species-detoxifying glutathione peroxidases and the H_2_O_2_-detoxifying enzyme catalase [40]. Similar antioxidant enzyme imbalances have been found in melanoma [41], as well as lung [42], prostate [43], and thyroid cancers [44]. Our microarray analysis determined that Ets-1 is a regulator of antioxidant gene expression in ovarian cancer cells, particularly glutathione peroxidases, which preferentially target H_2_O_2_ and lipid hydroperoxides for detoxification. This increased expression of antioxidants is in response to mitochondrial oxidative stress in the form of excessive reactive oxygen species production, and we have previously shown that Ets-1 gene expression increases in response to H_2_O_2_ [15]. However, it is important to note that Ets-1 also down-regulated genes encoding certain H_2_O_2_-detoxifying enzymes, suggesting that these cancer cells likely require and maintain a certain level of H_2_O_2_ to encourage the high growth rates inherent to tumour progression.

A novel function for Ets-1 was elucidated in this study following genomic and functional analysis of an ovarian cancer Ets-1 expression model. To our knowledge, this is the first study to show a role for this transcription factor in metabolism. Numerous down-regulated genes were identified in Ets-1 over-expressing cells including those encoding several mitochondrial proteins involved in oxidative phosphorylation, as well as important metabolic enzymes that are responsible for the generation of required substrates of the electron transport chain. Functional assays confirmed that Ets-1 over-expressing cancer cells displayed reduced oxidative phosphorylation capabilities, as well as enhanced reliance on glycolysis for cellular energy. Additionally, Ets-1 was shown to be important in the regulation of cellular O_2_ consumption further suggesting a reduced usage of oxidative phosphorylation in cancer cells expressing Ets-1.

Therefore, damage to the mitochondria results in increased production of H_2_O_2_ and consequent up-regulation of Ets-1, which then participates in an active regulatory network that encourages reliance on glycolysis and lipid metabolism for cellular energy requirements. Thus, Ets-1 may be grouped with other transcription factors that have been observed to up-regulate the expression of mitochondrial proteins (NRFs) or genes involved in glycolysis (HIF-1) in response to specific stresses [45], [46]. In summary, our findings demonstrate a novel role for Ets-1 in the regulation of cellular metabolism in response to mitochondrial stress.

## Materials and Methods

### Cell culture

The human ovarian carcinoma cell lines, 2008 and C13*, were kindly provided by Dr. Paul Andrews (Georgetown University, Rockville MD) [33]. The 2008 and C13* cells were maintained in RPMI 1640 medium supplemented with 10% fetal bovine serum and 2% penicillin/streptomycin. The stable cell line 2008-Ets1 [27] was maintained in growth medium as described with the addition of 200 ng/ml of the selective antibiotic Zeocin. All cells were kept at 37°C in a humidified atmosphere of 5% CO_2_. Media and supplements were purchased from Invitrogen Life Technologies (Burlington, ON, Canada), and FBS from Fisher Scientific (Ottawa, ON, Canada). All reagents were purchased from Sigma (Oakville, ON, Canada).

### Protein isolation and Western blot analysis

Whole cell lysates were collected, 30 µg of protein were separated by 10% SDS-PAGE electrophoresis, transferred to nitrocellulose membrane (Amersham Biosciences, Baie D'Urfe, QC, Canada), and blocked for 1 hr in 5% skim milk TBS-T. Membranes were incubated overnight with primary antibody in 0.5% skim milk TBS-T. Following primary antibody incubation, membranes were washed and incubated for 1 hr with horseradish peroxidase-linked IgG secondary antibody (1∶10,000) (Santa Cruz Biotechnology Inc., Santa Cruz, CA). Proteins were detected using ECL chemiluminescence reagent (Amersham), and exposed to film. Antibodies against Ets-1 (1∶100), G6PD (1∶1000), and PDHA (1∶500) were from Abcam; antibodies against Ets-2 (1∶500), PEA3 (1∶100), and HK (1∶250) were from Santa Cruz Biotechnology; and the antibody against CYC (1∶250) was from BD Biosciences.

### RNA isolation and quantitative real-time PCR

Total RNA was isolated using Trizol reagent as indicated by the manufacturer (Invitrogen). RNA samples were DNase treated using Turbo DNA-free™ as per the manufacturer's directions (Ambion), and 3 ug of total cellular RNA was reverse-transcribed using poly-T primers and the Superscript III First Strand Synthesis System (Invitrogen). Quantitative real-time PCR was conducted using a DNA Engine® thermal cycler (Bio-Rad) and Platinum SYBR Green qPCR SuperMix UDG (Invitrogen) with primer sequences listed in Table 5. Target gene expression was normalized to the pooled gene expression values of β-actin (ACTB), B-2 macroglobulin (B2M), glyceraldehyde-3-phosphdate dehydrogenate (GAPDH), and RNA polymerase II (RPII) as housekeeping controls. Data was normalized to housekeeper gene expression and efficiency corrected using the ΔΔCt method of relative quantification, where statistical significance and standard error were determined from ΔCt values.

**Table 5:** Primer sequences used for real time qRT-PCR.

Gene name (acronym)	Forward primer (5′-3′)	Reverse primer (5′-3′)
β-actin (ACTB)	cctccctggagaagagctac	gatgtccacgtcacacttca
B-2 macroglobulin (B2M)	gctatccagcgtactccaaag	tcacacggcaggcatactc
Glyceraldehyde-3-phosphate dehydrogenase (GAPDH)	atcatcagcaatgcctcctg	ctgcttcaccaccttcttga
RNA polymerase II (RPII)	gaaacggtggacgtgcttat	tctccatgccatacttgcac
Cytochrome *c* 1 (CYC1)	ccaatgaagatccccacatg	ccaggaaagtaggggttgaagt
V-ets erythroblastosis virus E26 oncogene homolog 1 (ETS1)	tgtattttgcatccctggtt	aacgacatcgattcaggact
Glucose-6-phosphate dehydrogenase (G6PD)	tggaaccgggacaacatc	caacaccttgaccttctcatcac
Glutathione peroxidase 2 (GPX2)	cccctacccttatgatgacc	gttgatggttgggaaggtg
Hexokinase 1 (HK1)	ggcgtttccacaagactcta	cttggtgaggtggaaatgag
Matrix metallopeptidase 13 (MMP13)	cagtctttcttcggcttagagg	cagaggagttacatcggacca
NADH dehydrogenase 1, α/β subcomplex 1 (NDUFAB1)	cgtctcgtcgtcctttcagc	cctggatgccctctaacg
Pyruvate dehydrogenase α 1 (PDHA)	cacagaccatctcatcacagt	ggcagacctcatcttttcca
Peroxisome proliferator-activated receptor γ (PPARG)	cctattgacccagaaagcgatt	cattacggagagatccacgga
Succinate dehydrogenase complex, subunit B (SDHB)	atcttgttcccgatttgagc	gtctccgttccaccagtacg

### Microarray analysis

Total RNA was isolated from tetracycline-induced 2008 and 2008-Ets1 cells as described, purified using the RNeasy purification kit (Qiagen), and hybridized to the GeneChip® Human Gene 1.0 ST Array (28,869 human gene probes). RNA quality analysis via bioanalyzer, microarray preparation, hybridization, and detection were performed by The Centre for Applied Genomics, The Hospital for Sick Children, Toronto, Canada. All low-level analyses, calculation of differential expression, and statistical adjustments were computed using R version 2.9.2 [47]. Assessment of RNA quality post-hybridization, and low-level analysis were performed using the package ‘affy’ [48]. RNA degradation plots revealed little 5′ to 3′ bias and all arrays were within 1.35 standard deviations of the average slope. Background correction and normalization was performed using the robust multi-array average (RMA) algorithm. To reduce the number of differential expression tests downstream, thereby increasing overall power, 50% of the lowest normalized Log_2_ probe-set intensities were removed according to recommendations by Hackstadt and Hess (2009) [49]. Differential expression estimates were computed using the adjusted Local Pooled Error test in the package ‘LPEadj’ [50]. The Benjamini-Hochberg procedure for controlling false discovery rate (FDR) was applied to comparison-wise p-values using the package ‘multtest’. Reported q-values represent the minimum FDR at which a particular test can be considered significant. Fold change expression values of 10 random target genes of varying fold change magnitude were validated using real time PCR. Corresponding relative fold changes were determined using the ΔΔCT method of relative quantification, and correlation coefficients were calculated to determine the relationship between real time PCR and microarray findings.

### Glycolytic dependency assays

Cellular growth rate and the inhibition of proliferation were evaluated by assessing total cell numbers, where 2×10^3^ cells were plated onto 96-well tissue culture plates and allowed to adhere. Cells were treated with 2-deoxy-D-glucose (2-DG; Sigma) administered at concentrations ranging from 1–7.5 mM over 96 hrs, at which time a Hoechst DNA content assay (Invitrogen) was performed. Following treatment, cells were washed and lysed in MilliQ water and 2 µg/mL Hoechst 33258 stain diluted in TNE buffer (10 mM Tris, 1 mM EDTA, 2 M NaCl, pH 7.4). Fluorescence was evaluated using a Cytofluor series 4000 multiwell plate reader (excitation 350 nm, emission 460 nm) (PerSeptive Biosystems, Framingham, MA). Cell number was standardized to fluorescence for each cell type by comparison with a standard curve of known cell numbers. To determine cell growth in glucose free media, glucose free RPMI and DMEM media supplemented with 110 mg/L pyruvate was added to cells following overnight adherence. Plates of cells were frozen every 24 hrs up to and including the 96 hr time point and Hoechst assays were performed at each time point.

### Oxygen consumption assay

All oxygen consumption assays were performed on the OROBOROS oxygraph (Oroboros, Innsbruck, Austria) at 37°C. Cells were pelleted, resuspended in growth medium, and 3×10^6^ cells were used per experiment at a density of 1×10^6^ cells/100 µL. Freshly harvested cells were added to the oxygraph chambers containing 1.8 mL of KCl medium (80 mM KCl, 10 mM Tris-HCl, 3 mM MgCl_2_, 1 mM EDTA, 5 mM potassium phosphate, pH 7.2). The total O_2_ concentration and flux were recorded at 1 sec intervals throughout the experiment. Once the oxygen concentration stabilized, the basal O_2_ consumption was determined for each cell line and condition, where OROBOROS software was used for data acquisition and analysis.

### Statistical analysis

Data is presented as the mean +/− standard deviation from at least three independent experiments. Statistically significant differences between sample groups were determined using a Student's t-test or ANOVA where applicable, with a p-value≤0.05 considered to be statistically significant (p≤0.05 = *, p≤0.01 = **, p≤0.001 = ***).
